# Epidemiological investigation of a large trichinellosis outbreak in Lebanon, October 2023–February 2024

**DOI:** 10.1017/S0950268826101885

**Published:** 2026-06-29

**Authors:** Hawraa Sweidan, Jeanne El Hage, Nadine Omeirat, Lina Chaito, Nada Ghosn

**Affiliations:** 1 Epidemiological Surveillance Programme, Lebanon Ministry of Public Health, Lebanon; 2 Mediterranean and Black Sea Programme in Intervention Epidemiology Training (MediPIET), European Centre for Disease Prevention and Control, Sweden; 3 Animal Health Laboratory, Lebanese Agricultural Research Institute, Fanar-Lebanon, Lebanon; 4 American University of Beirut Medical Center, Lebanon

**Keywords:** Trichinellosis, Trichinella spiralis, foodborne outbreak, Lebanon, meat consumption, zoonotic disease, food safety, One Health

## Abstract

On 4 December 2023, trichinellosis cases were notified in Jahliyeh, Mount Lebanon. We investigated to determine the source, magnitude, and control measures. We defined a suspected case as a Jahliyeh resident with ≥1 compatible symptom diagnosed by a physician after 1 October 2023; a probable case as having ≥3 typical symptoms (fever, myalgia, edema, or eosinophilia); and a confirmed case as a suspected or probable case with laboratory confirmation by muscle biopsy or serology. We surveyed reachable households (252/561) to identify cases and assess meat consumption practices, inspected local butcheries, and tested meat samples. We identified 290 cases: 268 (92%) suspected, 21 (7.2%) probable, and 1 (0.3%) confirmed. Median age was 35 years (range 2–85); 34% required hospitalization. Illness was associated with Butchery A meat (risk ratio [RR] = 8.2; 95% confidence interval [CI]: 5.6–12.1) and uncooked meat (RR = 1.9; 95% CI: 1.4–2.6), while freezing and cooking were protective. Food inspection revealed major hygiene breaches at Butchery A and *Trichinella* larvae in minced beef and sausage. This outbreak was epidemiologically confirmed despite limited laboratory confirmation in humans. We recommend strengthening food safety inspections, intersectoral One Health collaboration, laboratory capacity, and public awareness of safe meat preparation.

## Introduction

Trichinellosis is a zoonotic parasitic disease caused by parasitic roundworms (nematodes) of the genus *Trichinella*, can be transmitted from animals to humans [1]. The genus currently comprises at least 10 recognized species and 3 additional genotypes that have not yet been formally classified, reflecting substantial genetic and ecological diversity [2]. Numerous animal species serve as reservoirs for human infection, with prevalent hosts such as pigs, wild boars, rodents, bears, and horses. While *Trichinella spiralis* is the species most frequently associated with human infections and has been identified in all regions of the world, other species and genotypes – including *T. nativa*, *T. britovi*, and *T. pseudospiralis* – are geographically widespread and maintained primarily in wildlife reservoirs across Europe, Asia, Africa, and the Americas [2].

Human infection occurs through the consumption of inadequately cooked or raw meat and meat products containing the parasite’s larvae [3], triggering a pathogenesis that unfolds in a biphasic course: the enteral and parenteral phase. During the first week (enteral phase), the penetration of the intestinal mucosa may cause mild, transient gastrointestinal distress – such as nausea, vomiting, and abdominal pain, though many cases remain asymptomatic or are misdiagnosed as common food poisoning. As the disease progresses into the parenteral phase (weeks 2 through 6), symptoms shift from intestinal to systemic as newborn larvae migrate throughout the body and penetrate muscle and other tissues. This stage is clinically characterized by periorbital edema, high fever, and diffuse myalgia, though severe infections can lead to more critical complications, including myocarditis, pneumonia, or neurological manifestations like encephalitis. While acute symptoms typically diminish by the sixth week of convalescence, the larvae successfully establish themselves within muscle ‘nurse cells’, where they can persist for years, potentially undergoing eventual calcification [4]. Additionally, severe infections can be complicated by life-threatening cardiovascular, pulmonary, or neurologic involvement, including myocarditis, pneumonia, and encephalitis [5]. The global burden of trichinellosis has been estimated at approximately 76 disability-adjusted life years (DALYs) per billion persons per year (95% confidence interval: 38–129) reflecting a low overall burden at the global level [6].

In Lebanon, sporadic cases of trichinellosis have been reported in past years, typically linked to the consumption of hunted wild boar meat [7]. This regional pattern is exemplified by an outbreak in Izmir, Turkey, which demonstrated that large-scale clinical events could occur when contaminated meat enters the food chain [8]. In 2022, the Epidemiological Surveillance Unit (ESU) of the Ministry of Public Health of Lebanon (MoPH) investigated an outbreak in the Koura district. Eighteen cases were detected and epidemiologically linked to the consumption of raw meat labelled as ‘goat meat’. The meat was found to be contaminated with *Trichinella* spp., likely due to cross-contamination with utensils used to cut hunted boar meat, that was illicitly procured and mixed with the legitimate meat products, highlighting ongoing gaps in food safety and meat inspection practices.

On 5 December 2023, a university hospital contacted the ESU to report a suspected case of trichinellosis. The patient was from Jahliyeh, a town in Chouf district, Mount Lebanon, with approximately 6000 residents.

One day earlier, the municipality of Jahliyeh had reported to the ESU call centre an increase in gastroenteritis cases. The ESU initiated an investigation to confirm the outbreak of trichinellosis, assess its magnitude, determine the sources, evaluate food safety in local butcheries, and guide the implementation of necessary preventive measures.

## Methods

The outbreak investigation included setting case definition, active and passive case finding, a household survey, meat investigations, and a retrospective cohort analysis based on data collected during the household survey.

### Case definition

We defined a suspected case as any resident of Jahliyeh with a history of meat consumption in the district who developed at least one symptom suggestive of trichinellosis, such as fever or edema, and was diagnosed by a physician between 1 October 2023 and 28 February 2024. We defined a probable case as a suspected case presenting with at least three symptoms indicative of trichinellosis, including fever, myalgia, edema, or eosinophilia (>500 eosinophils/mm^3^), within the same period. We defined a confirmed case as a suspected or probable case with laboratory confirmation through a *Trichinella*-positive muscle biopsy, a positive serological result using immunofluorescence assay (IFA) or other validated serological tests. Case definitions were adapted from CDC guidelines [9]. These definitions were adjusted to prioritize clinical and epidemiological criteria due to the local unavailability of commercial Trichinella serological assays during the investigation. This adaptation was necessary to ensure a comprehensive assessment of the outbreak’s magnitude in a resource-limited setting, where relying solely on laboratory-dependent criteria would have resulted in a significant underestimation of the public health impact.

### Case finding methods

The case finding included enhancing passive surveillance, conducting active surveillance, and a household survey to assess the magnitude of the outbreak.

### Passive surveillance

We enhanced passive surveillance by informing focal points in health facilities across the Chouf District about the potential outbreak and providing them with the case definition. Cases could be reported through the District Health Information Software 2 (DHIS2) online reporting form or via the 1787 hotline. Passive surveillance in this context refers to routine reporting of cases by healthcare providers without additional outreach or active case searching.

### Active surveillance

On 12 and 13 December 2023, we visited local and neighbouring health facilities in the town, including medical centres, primary healthcare centres, and hospitals. During these visits, we systematically checked logbooks for patients who had sought medical care with symptoms and signs compatible with trichinellosis. We also interviewed patients attending health facilities who presented compatible symptoms. On 12 and 13 December 2023, we visited the primary healthcare centre to collect data on patients referred by physicians for blood tests or prescribed anthelmintic medication. We contacted focal points at neighbouring hospitals to report probable or suspected cases among Jahliyeh residents. Active surveillance involved direct case finding by the outbreak investigation team through household visits, interviews, and review of health facility records.

### Household survey

To assess the extent of the outbreak and identify its source, we surveyed households in the village. We obtained a phone registry from local authorities and attempted to interview the head of each household. We defined a household as a group of individuals residing in the same dwelling and sharing living arrangements, including both family members and non-family residents. Eligible households were those located within the administrative boundaries of Jahliyeh and with at least one member present during the outbreak investigation period. Households listed in the municipal registry but without residents at the time of the investigation were excluded. We attempted to contact all households in the village.

### Data collection

To interview individual cases identified by passive and active surveillance, we used an online questionnaire created using the Survey123 app, developed by Esri [10]. The questionnaire could be deployed on mobile devices, allowing offline data collection. We compiled and managed the forms collected through the DHIS2 platform [11].

For the household survey, we collected information on the household and its residents from an adult responding to the call or another designated adult who was residing in the household and could provide information on its residents. The interviewers recorded responses on the District Health Information Software 2 (DHIS2) platform.

For each household, we collected household-specific data, including the type of meat purchases (cow/sheep or pork), frequency of purchase per month, source of purchase (local butcher shops, supermarkets, direct from farms, hunted or trapped animals, or other markets), meat storage (refrigerator or freezer), preparation practices (unprocessed, marinated, frozen, smoked, ground, dried jerky, or other), cooking methods (uncooked, fried, boiled/oven, or barbecue), and cooking level (well done or undercooked). However, the quantity of meat consumed was not systematically recorded. Additionally, we collected information on consumption by each resident, that is, whether they consumed meat along with the family, or had different habits, such as being vegetarian. Based on the information on each resident, we were able to identify residents meeting suspected case definition criteria, and their meat consumption habits.

### Data analysis

We first conducted univariable analyses to calculate attack rates (ARs) and risk ratios (RRs) with 95% confidence interval (CI) for each exposure. This allowed us to assess the association between individual exposures and illness.

To examine independent associations, we then applied multivariable logistic regression analysis. We initially ran univariable logistic regression for each exposure to estimate unadjusted odds ratios (ORs). Exposures with a *p*-value <0.05 in the univariable logistic models were considered candidates for the multivariable model. The final multivariable logistic regression model provided adjusted odds ratios (aOR) with 95% CI, reflecting the independent effect of each exposure after adjustment. All analyses were performed using R software (version 4.5.0).

### Meat source investigations

On 6 December 2023, the ESU inspected the two local butcheries (A and B). The butcheries were inspected for hygiene standards, meat storage conditions, sourcing documentation, and compliance with public health regulations.

The inspectors collected leftover food samples from households and the two butcheries and transported them in sterile containers with icepacks to the laboratory. The Lebanese Agriculture Research Institute (LARI) tested the specimens for *Trichinella* sp. infection and Salmonella contamination.

The laboratory used the digestion method using the magnetic stirrer [12]. A minimum of 50 g of muscle tissue was used from each sample. The tissue was chopped up in a blender and transferred to 1 l of digestion fluid (acid solution with pepsin) kept at a constant temperature of 44–46°C for 30 min. After digestion, the digestion fluid was poured through a 180 μm sieve into a separation glass funnel and left undisturbed for 30 min: The *Trichinella* larvae settled at the bottom of the funnel. 60 ml of digestion was collected and washed. Immediately after the washing steps, a stereomicroscope was used at a 15 to 20× magnification to examine the samples.

### Ethical considerations and consent

We did not apply for ethical approval of our investigation since surveillance and investigation of outbreak of communicable diseases are exempted by law from the requirement for ethical clearance. We obtained oral informed consent from the designated household heads on behalf of all residents before proceeding with the interviews. During this process, we clearly explained the purpose of the investigation and the voluntary nature of their participation. To ensure the confidentiality of all subjects, names were anonymized and replaced with unique identification codes for data analysis. The data collected from case subjects and household residents, including the list of patients, were stored securely on DHIS2, the Ministry’s password-protected and secure database, to which only authorized data collectors had access.

## Results

A university hospital reported on 5 December 2023, a clinical report about a 34-year-old male patient, previously healthy, presented with a 3-day history of high-grade fever (up to 39.5°C), severe myalgia, and generalized fatigue. Ten days prior to presentation, he had consumed raw kibbeh and sheep brain. Within 2 days of ingestion, he developed watery diarrhoea that improved after a short course of metronidazole; however, he subsequently developed fever and severe myalgias and fatigue, prompting hospital admission on 1 December 2023.

On examination, he was febrile (39.5°C), haemodynamically stable, and had a faint maculopapular rash over the lower extremities and abdomen. Neurological and abdominal exams were unremarkable. Laboratory evaluation revealed leucocytosis (12800/μl) with marked eosinophilia (22%), elevated CRP (101 mg/L), mildly disturbed transaminases, and hyponatremia. Abdominal imaging was unremarkable. Blood cultures and serological testing for Strongyloides, Fasciola, *E. granulosus*, and Toxocara were negative.

The patient started empirically on albendazole (400 mg PO BID) given the suspicion of Trichinellosis. On day 5 of hospitalization, prednisone (60 mg PO daily) was initiated for persistent systemic symptoms and worsening eosinophilia. A muscle biopsy on day 6 revealed multifocal granulomatous myositis, with rare sections showing a parasite morphologically consistent with *Trichinella* spp., confirming the diagnosis.

Following the report of the suspected Trichinellosis index case on 5 December 2023, retrospective investigation identified a total of 290 cases with onset between weeks 44 and 52 of 2023. Based on case classification, 92.4% (*n* = 268) were suspected, and 7.2% (*n* = 21) were probable. Only the index case, notified on 8 December 2023, was confirmed by histopathology. This patient reported that symptom onset occurred on 22 November 2023.

**Figure 1.**
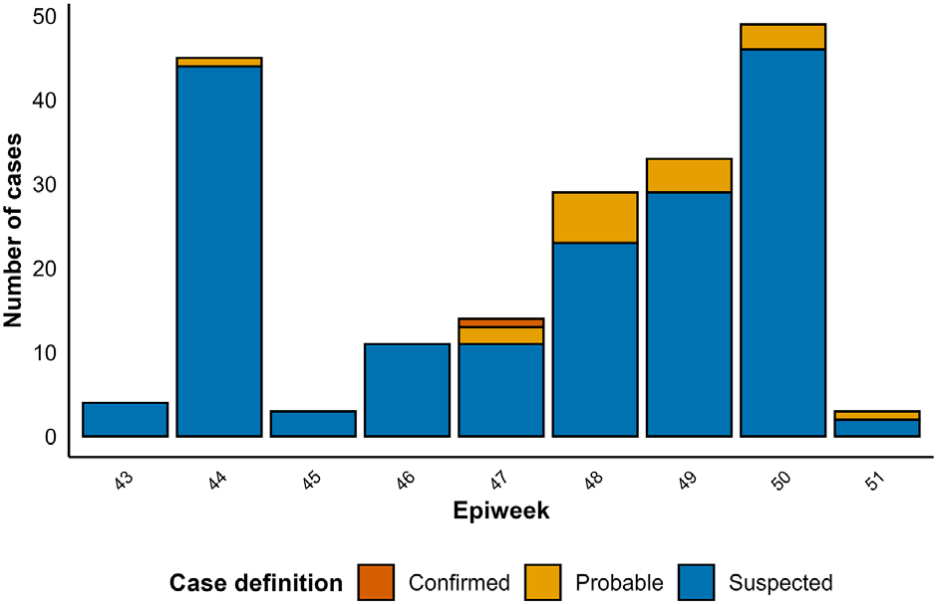
Trichinellosis cases by week of onset, October 2023–February 2024. Figure 1. long description.

The epidemic curve (Figure 1) indicated an intermittent common-source outbreak, with the initial peak of illness onset observed in week 44 and another peak in week 50. This pattern suggested that the population was exposed to the contaminated source on two separate occasions.

Among the 290 cases, one was reported by a hospital, 167 detected by active search in primary health care centres and hospitals, and 112 identified during the household survey.

The sex ratio (M:F) was 0.9 (137:153), with females constituting a slightly higher proportion of cases (52.8%). Cases were distributed across all age groups, however; most cases fell within the 10–59 age range, which accounted for more than 75% of cases. Hospital admission was required for 34.1% of cases (Table 1).

**Table 1.** Demographic and clinical characteristics of trichinellosis cases, Jahliyeh, Lebanon, 2023 (*N* ^a^ = 290) Table 1. long description.

Characteristic		Group	Count	Valid %
Age group		0–9	16	5.7
		10–19	61	21.8
		20–29	41	14.6
		30–39	40	14.3
		40–49	40	14.3
		50–59	46	16.4
		60–64	22	7.9
		65+	14	5.0
		Missing	10	–
Sex		Female	153	52.8
		Male	137	47.2
Hospital admission		No	145	65.9
		Yes	75	34.1^b^
		Missing	70	–
Total				290

*Note:* Percentages calculated excluding missing values.

a *N* includes suspected, probable, and confirmed cases of trichinellosis.

b Valid percentage, calculated by excluding the 70 cases with missing data.

The most frequently reported symptoms were myalgia (92.3%), followed by fever (80.3%) and abdominal pain (72.4%) (Table 2). The most frequently reported symptoms were myalgia (92.3%), followed by fever (80.3%) and abdominal pain (72.4%) (Table 2). Gastrointestinal symptoms were common, with abdominal pain frequently co-occurring with diarrhoea (53.0%) and nausea (62.1%), while systemic and dehydration-related symptoms such as periorbital oedema (64.9%), sweating (53.2%), and thirst (40.0%) were also frequently reported. Other common symptoms included periorbital oedema (64.9%), nausea (62.1%), sweating (53.2%), and diarrhoea (53.0%). Regarding meat preparation practices, the largest proportion of cases reported consuming uncooked meat (36.2%), followed by barbeque (24.1%) and fried meat (16.6%) (Table 2).

**Table 2.** Distribution of symptoms and meat preparation methods among trichinellosis cases, Jahliyeh, Lebanon, 2023 (*N* ^a^ = 290) Table 2. long description.

Characteristic	Group	Count	Valid %
Symptoms	Myalgia	239	92.3
	Fever	191	80.3
	Abdominal pain	147	72.4
	Periorbital oedema	131	64.9
	Nausea	121	62.1
	Diarrhoea	116	53.0
	Sweating	99	53.2
	Thirst	72	40
	Photophobia	63	35
Meat preparation	Uncooked	105	36.2
	Barbecue	70	24.1
	Fried	48	16.6

a *N* includes suspected, probable, and confirmed cases of trichinellosis.

Table 3 shows that hospitalized cases experienced a higher frequency of multiple symptoms compared with non-hospitalized cases, particularly myalgia (94.4% vs. 85.9%), abdominal pain (78.8% vs. 70.4%), and diarrhoea (71.0% vs. 63.0%).

**Table 3.** Distribution of symptoms by hospital admission status Table 3. long description.

Symptom	Hospitalized *n*/*N* (%)	Not hospitalized *n*/*N* (%)
Myalgia	34/36 (94.4%)	61/71 (85.9%)
Abdominal pain	26/33 (78.8%)	50/71 (70.4%)
Fever	26/35 (74.3%)	61/74 (82.4%)
Diarrhoea	22/31 (71%)	46/73 (63%)
Periorbital oedema	17/24 (70.8%)	34/59 (57.6%)
Sweating	18/26 (69.2%)	26/62 (41.9%)
Nausea	15/25 (60%)	44/66 (66.7%)
Photophobia	15/26 (57.7%)	22/63 (34.9%)
Thirst	12/23 (52.2%)	17/62 (27.4%)

*Note: n* represents the number of cases with the symptom; *N* represents the total number of cases in that category with available symptom-specific data. Denominators vary due to incomplete clinical records for some patients.

### Household survey

Out of the 762 households in the local registry, 561 were eligible (correct phone number and still residing in Jahliyeh), of which 252 (45%) completed the survey including 984 residents. Out of 252 households surveyed, household sizes ranged from 1 to 11 members, with a median of 4. Among these, 43 households had at least one symptomatic member, while 163 households reported no symptoms.

The univariable analysis (Table 4) showed that consumption of meat from butchery A and eating uncooked meat were associated with higher risk of illness, with relative risks of 8.21 and 1.93, respectively. In contrast, several factors appeared protective, including purchasing meat from other markets (RR = 0.32), storing meat in the freezer (RR = 0.53), freezing meat before consumption (RR = 0.36), and using cooking methods such as frying (RR = 0.57) or oven/boiling (RR = 0.50). These factors were associated with lower attack rates and reduced relative risks.

**Table 4.** Univariable analysis of food-related exposures and risk of trichinellosis in the household survey, Jahliyeh, Lebanon, 2023 (*N* = 984) Table 4. long description.

Category	Risk factor	Exposed				Not Exposed						
		Ill	Not ill	Total	Attack rate	Ill	Not ill	Total	Attack rate	Relative risk	95% CI lower	95% CI upper
Meat source	Butchery A	107	132	239	0.448	27	476	503	0.05	8.21	5.56	12.12
	Other Market	46	416	462	0.1	50	110	160	0.31	0.32	0.22	0.46
Storage area	Freezer	37	265	302	0.123	97	322	419	0.23	0.53	0.38	0.75
Preparation method	Frozen	13	155	168	0.077	120	429	549	0.22	0.36	0.21	0.62
Cooking method	Uncooked	66	180	246	0.268	68	423	491	0.14	1.93	1.43	2.61
Cooking method (if cooked)	Fried	78	439	517	0.151	56	157	213	0.26	0.57	0.42	0.78
	Oven (boiled)	109	546	655	0.166	25	50	75	0.33	0.5	0.35	0.71

*Note:* CI = confidence interval.

### Multivariable analysis

In the multivariate logistic regression (Table 5), consumption of uncooked beef products from butchery A was significantly associated with illness (aOR = 12.69), with individuals having 12.7 times higher odds of infection compared to other sources. Similarly, individuals who reported consuming uncooked meat had 2.57 times higher odds of developing the illness compared to those who did not consume uncooked meat (aOR = 2.57). In contrast, proper meat handling and preparation methods – including freezing, and oven/boiled cooking – were significantly protective, reducing the likelihood of illness by 44–69%. Consumption of fried meat showed no significant association with illness.

**Table 5.** Multivariable logistic regression of meat consumption practices in the household survey, Jahliyeh, Lebanon, 2023 Table 5. long description.

Variable	aOR	95% CI lower	95% CI upper
	0.074	0.033	0.168
Butchery A	12.690	7.777	20.708
No further processing	1.877	1.066	3.303
Frozen	0.234	0.119	0.462
Oven/boiled	0.382	0.195	0.751
Uncooked	2.570	1.556	4.246
Fried	0.847	0.512	1.403

*Note:* aOR = odds ratio adjusted to other covariates; CI = confidence interval.

### Food safety inspection

On 6 December 2023, the ESU team conducted a food safety inspection at butcheries A and B using a standardized checklist. The scores are presented in Table 6. While the inspection of butchery A revealed inadequate hygiene practices, improper meat storage, and other non-compliant procedures that resulted in significant food safety breaches, these findings are not specific factors for *Trichinella* transmission. More relevant to the outbreak investigation were gaps in meat sourcing documentation and traceability, which limited the ability to identify the origin of the implicated products. Information on meat sourcing relied on self-reporting by the seller and could not be independently verified.

**Table 6.** Findings from the food inspection checklist for butcheries, Jahliyeh, Lebanon, 2023 Table 6. long description.

Section	Butchery A	Butchery B	Expected score
Food preparation	5.5	12.0	18
Employees and food handlers	1.0	1.5	11
Food handling and preparations	8.0	9.5	22
Disinfection and cleaning	0.0	1.5	3
Restrooms and changing rooms	2.5	5.5	13
Sanitary facilities	4.0	4.5	17
Total	21.0	34.5	84

### Food testing

Food safety inspectors collected a total of 11 food samples. One of these samples was collected as leftovers from households, while the remaining were sourced from the two local butcheries: four from butchery A and six from butchery B. Laboratory analysis at LARI identified *Trichinella* spp. larvae in two samples – minced beef and beef sausage – both sourced from Butchery A. Based on the laboratory results, the butchery (A) was closed upon the request of the Ministry of Public Health.

## Discussion

This investigation describes a large-scale epidemiologically confirmed trichinellosis outbreak in Jahliyeh, Mount Lebanon, characterized by widespread exposure, a high proportion of hospitalisations, and laboratory confirmation of Trichinella larvae in implicated meat products. A total of 290 cases were linked to the consumption of contaminated meat labelled as beef, from a single, non-compliant local butchery. Although human laboratory confirmation was limited to a single case confirmed by muscle biopsy and histopathology, the convergence of clinical presentation, epidemiological links, and food laboratory findings provides strong evidence supporting the diagnosis of trichinellosis. By international standards, an outbreak is defined as an increase in cases above the expected background level for a specific area and period. Given the rarity of trichinellosis in this region and the clear clustering of 290 symptomatic residents linked to a confirmed contaminated source, this event meets the technical and functional definition of a large-scale outbreak, regardless of the proportion of cases confirmed via invasive biopsy. Human infection is most often associated with consumption of wild game, particularly wild boar, which serves as a reservoir for *Trichinella* spp. [13]. This event constitutes one of the largest reported *Trichinella* outbreaks in Lebanon in the last decade [6], signalling a failure in national meat safety controls. The size of the outbreak and the 34% hospitalization rate underscore the severity of the illness and the burden placed on local healthcare services. Crucially, the magnitude of this outbreak may have been exacerbated by the economic and financial crises in Lebanon, that may have affected food safety and food security [14].

This outbreak was likely caused by contaminated meat labelled as beef but likely contaminated with wild boar meat, sold by butchery A. Given that butchery A was reportedly authorized only to handle beef and lamb, the detection of *Trichinella* spp. in their products points to a likely mixing of beef products with illicit meat from an undisclosed source. Trichinellosis infections are rarely linked to meat from commercially raised pigs; since pigs kept under controlled housing conditions have a negligible risk, in contrast to pigs under non controlled housing conditions. Typically pigs from non-controlled holdings and carnivorous or omnivorous wildlife intended for human consumption pose a risk [15, 16]. ESU investigated a similar outbreak in 2022, where mixing of legitimately tested and illicitly procured untested meat, led to trichinellosis cases. A recent investigation pointed to frequent use of unregulated wild boar hunting as a food source in Lebanon, due to an ongoing economic crisis [17]. We could not confirm this hypothesis nor trace the possible source of contamination, because the butcher denied any involvement in such practices and refused further collaboration.

More than one-third of residents of Jahliyeh reported consuming raw meat during the exposure period. Consumption of raw meat is deeply rooted in local cultural traditions. A similar pattern was observed during the 2022 outbreak in Koura district, where raw meat, served as a traditional dish at festive gatherings, was implicated. A survey (*n* = 577) of Lebanese adults found 74.5% reported consuming raw meat. The study further revealed significant deficits in risk awareness: only 44% had good food safety knowledge. The combination of high-risk cultural food practices and significant deficits in consumer knowledge and safe handling behavior create the foundational conditions for recurrent Trichinellosis outbreaks across the country [17].

During this outbreak, a rapid response involving collaboration between the Ministry of Public Health (MOPH), the Ministry of Agriculture (MoA), the Ministry of Interior Affairs (MoIA), and local authorities was essential for containment. The MoIA was responsible for the legal closure of the implicated source (butchery A) after the results of the food inspection were communicated by the MOPH, the MoA provided necessary laboratory capacity (LARI) and testing for the meat samples, and the MOPH coordinated case management, medication supply, and community outreach. As demonstrated in a Canadian outbreak, a rapid, unified response involving public health, laboratory, and enforcement sectors is vital not only for outbreak containment but also for maintaining public trust within the affected community [18].

During this outbreak, molecular diagnostic capacity was not available in the country, and laboratory confirmation was restricted to a single case confirmed via muscle biopsy and histopathology. Even though serological testing is another valuable diagnostic tool, it was not available at the local level in Lebanon. Commercial serological assays, such as enzyme-linked immunosorbent assays (ELISA) and Western blot, have been widely used internationally for surveillance and outbreak confirmation [19]. This insufficient laboratory capacity limits timely case management and epidemiological investigation. In recent years, rapid diagnostic tests became available, such as highly sensitive and specific immunochromatographic tests (ICT) [20]. The deployment of such tests could significantly improve future detection rates and enhance the overall surveillance strategy, reducing dependence on lengthy, invasive procedures like biopsy.

### Limitations

First, only a portion of households responded to the survey, with an estimated response rate of 45%. This low participation may have introduced non-response bias and affected the representativeness of our findings, as non-responding households might also have consumed meat from butchery A, potentially leading to an underestimation of the attack rate. Second, recall bias was likely because information on food consumption and symptoms was collected retrospectively. Participants may have underreported or misreported exposures, particularly for minor or asymptomatic cases, which could have influenced the observed associations and the calculated attack rates. Third, underreporting may have occurred among individuals who sought care from private physicians rather than public health facilities. Despite follow up with the private clinicians, those cases were most likely not captured, leading to an underestimation of disease burden. Fourth, laboratory confirmation was limited. Only one case was confirmed via histopathology, and serological testing was unavailable in Lebanon. The laboratory capacity constraints restricted the ability to confirm additional cases and perform more detailed diagnostic investigations.

## Conclusions

The epidemiologically confirmed trichinellosis outbreak in Jahliyeh in October–December 2023 was linked to contaminated meat labelled as beef from a local butcher. The investigation pointed to clandestine butchery practices and non-compliance with meat handling regulations. All affected individuals reported consuming raw or undercooked meat, particularly in traditional dishes, which facilitated the transmission of the parasite. The investigation and the multi-approach implementation of the control measures lead to stopping the outbreak.

### Recommendations

Following the outbreak, −short-term measures-immediate actions were implemented to contain the event. The implicated butcher shop was closed. Identified cases received management at local primary healthcare centres according to the national protocol. Albendazole was provided by the Ministry of Public Health and distributed to the primary healthcare centre in the district for patient treatment. Community awareness sessions and informational brochures were distributed to educate residents about trichinellosis, its transmission, and prevention.

To prevent future outbreaks, it is essential to strengthen food safety systems in line with the WHO Global Strategy for Food Safety 2022–2030 [21], as well as international guidelines and recommendations from WHO, FAO, and WOAH. This includes enhancing regulatory oversight of slaughterhouses, butcher shops, and food markets, enforcing systematic meat inspection, including additional testing of meat samples from susceptible slaughtered animal species such as pigs, horses, and wild boar, and ensuring traceability from source to consumer. Public health campaigns should raise awareness about the risks of consuming raw or undercooked meat and promote safe preparation methods that maintain traditional flavours. Surveillance and laboratory capacity should be improved through a One Health approach, integrating human and animal health sectors, equipping laboratories for serological and histopathological testing, and combining active case finding with routine surveillance to detect asymptomatic or mild cases. These measures provide both immediate containment and sustainable strategies to reduce the risk of trichinellosis and other foodborne illnesses. Finally, a One Health approach is needed to combine different sectors, such as public health and veterinary public health to strengthen the surveillance and response strategies [22]. Ensuring laboratories are equipped to perform the necessary diagnostic tests will facilitate timely detection, prevention, and control of zoonotic diseases, ultimately reducing the risk of future outbreaks.

## Data Availability

The data that support the findings will be available in Trichinellosis-outbreak-Lebanon at https://github.com/Haouurra/Trichinellosis-outbreak-Lebanon following 1 month from the date of publication.

## Figure 1 Long description

A bar chart titled ‘Epidemic Curve of Trichinellosis Cases, Jahliyeh, Lebanon, 2023’. The horizontal x-axis shows the week of illness onset, spanning from week 44 to week 52 of 2023. The vertical y-axis shows the number of cases. Two distinct peaks are visible: the first and larger peak occurs at week 44, and a second smaller peak occurs at week 50, indicating an intermittent common-source outbreak with exposure on two separate occasions. The remaining weeks show lower case counts between and after the two peaks.

Navigate back to Figure 1.

## Table 1 Long description

Table 1 presents demographic and clinical data for 290 trichinellosis cases. Age group breakdown: 0–9 years: 16 cases (5.7%); 10–19: 61 (21.8%); 20–29: 41 (14.6%); 30–39: 40 (14.3%); 40–49: 40 (14.3%); 50–59: 46 (16.4%); 60–64: 22 (7.9%); 65 and over: 14 (5.0%); 10 missing. Sex: 153 female (52.8%), 137 male (47.2%). Hospital admission: 145 not admitted (65.9%), 75 admitted (34.1%), 70 missing. Percentages exclude missing values.

Navigate back to Table 1.

## Table 2 Long description

Table 2 shows symptoms and meat preparation among 290 cases. Symptoms (valid %): Myalgia 92.3%, Fever 80.3%, Abdominal pain 72.4%, Periorbital edema 64.9%, Nausea 62.1%, Diarrhoea 53.0%, Sweating 53.2%, Thirst 40%, Photophobia 35%. Meat preparation method: Uncooked 105 cases (36.2%), Barbecue 70 (24.1%), Fried 48 (16.6%). Cases include suspected, probable, and confirmed trichinellosis.

Navigate back to Table 2.

## Table 3 Long description

Table 3 compares symptom prevalence between hospitalized and non-hospitalized cases. Hospitalized vs. not hospitalized: Myalgia 94.4% vs. 85.9%; Abdominal pain 78.8% vs. 70.4%; Fever 74.3% vs. 82.4%; Diarrhoea 71.0% vs. 63.0%; Periorbital oedema 70.8% vs. 57.6%; Sweating 69.2% vs. 41.9%; Nausea 60.0% vs. 66.7%; Photophobia 57.7% vs. 34.9%; Thirst 52.2% vs. 27.4%. Denominators vary per symptom due to incomplete clinical records. N represents total cases in each category with available symptom-specific data.

Navigate back to Table 3.

## Table 4 Long description

Table 4 presents univariable analysis for 984 participants. For each risk factor, counts of ill and not-ill individuals are shown for exposed and unexposed groups, with attack rates and relative risk (RR) with 95% confidence intervals. Purchasing from Butchery A showed the highest risk: attack rate 44.8% exposed vs. 5% unexposed, RR 8.21 (95% CI 5.56–12.12). Other market showed protective effect: RR 0.32 (95% CI 0.22–0.46). Freezer storage: RR 0.53 (0.38–0.75). Frozen preparation: RR 0.36 (0.21–0.62). Uncooked consumption: RR 1.93 (1.43–2.61). Fried cooking: RR 0.57 (0.42–0.78). Oven/boiled: RR 0.50 (0.35–0.71).

Navigate back to Table 4.

## Table 5 Long description

Table 5 shows multivariable logistic regression results with adjusted odds ratios (aOR) and 95% confidence intervals. Intercept: aOR 0.074 (0.033–0.168). Butchery A: aOR 12.690 (7.777–20.708), the strongest risk factor. No further processing: aOR 1.877 (1.066–3.303). Frozen preparation: aOR 0.234 (0.119–0.462), protective. Oven/boiled cooking: aOR 0.382 (0.195–0.751), protective. Uncooked consumption: aOR 2.570 (1.556–4.246), increased risk. Fried cooking: aOR 0.847 (0.512–1.403), not statistically significant.

Navigate back to Table 5.

## Table 6 Long description

Table 6 compares inspection scores for Butchery A (implicated) and Butchery B across six categories, with maximum expected scores. Food preparation: Butchery A 5.5, Butchery B 12.0, expected 18. Employees and food handlers: A 1.0, B 1.5, expected 11. Food handling and preparations: A 8.0, B 9.5, expected 22. Disinfection and cleaning: A 0.0, B 1.5, expected 3. Restrooms and changing rooms: A 2.5, B 5.5, expected 13. Sanitary facilities: A 4.0, B 4.5, expected 17. Total scores: Butchery A 21.0, Butchery B 34.5, out of a maximum of 84. Both butcheries scored well below the expected total, with Butchery A scoring markedly lower.

Navigate back to Table 6.
